# Pulmonary embolism and patent foramen ovale thrombosis: the key role of TEE

**DOI:** 10.1186/1476-7120-5-26

**Published:** 2007-08-24

**Authors:** Walter Serra, Giuseppe De Iaco, Claudio Reverberi, Tiziano Gherli

**Affiliations:** 1 Heart department, Cardiology division, Azienda Ospedaliera/Universitaria Parma, Italy; 2 Heart department, Cardiac heart surgery institute, Azienda Ospedaliera/Universitaria Parma, Italy

## Abstract

This is a case report of a 35 young man with Klinefelter Syndrome presented breathlessness, palpitations and chest pain. It shows a rare case of a thrombus located through the PFO, in patient with pulmonary and paradoxical embolism, which takes back to exciting hypothesis on thrombus growth. A thrombus, which has grown 'in situ' or trapped through the patent foramen ovale, may be a cause of relapsing pulmonary or systemic embolism during anticoagulation therapy.

To prevent recurrent paradoxical embolism, percutaneous closure of PFO is recommended, but in this case, thrombus was trapped through the PFO and the patient was referred to the surgeon.

We believe that under these circumstances the clinician should be informed of the presence of PFO in critical pulmonary embolism; this case points out the key role of TEE to face a diagnostic and therapeutic scenarios.

## Background

In 1942 Klinefelter published a report of 9 men that were tall, with a eunuch habit, small testicles and with gynecomastia [1]. In 1959 these patients with Klinefelter Syndrome phenotype were discovered to have genotype XXY.

The cardiac malformation and cardiovascular disease associated with this syndrome are reported below:

Mitral valve prolapse occurs in 55% of patients. Varicous veins in 20–40%.

Prevalence of vein ulcerations is 20% greater than normal population.

High risk of deep vein thrombosis and pulmonary embolism.

Imaging plays a key diagnostic role in context of a correct management of chest pain with an increase of serum and electrocardiogram alterations.

## Case presentation

A 35 years old man with Klinefelter Syndrome was admitted to hospital with a two-week history of progressive breathlessness, palpitations and chest pain.

Previous saphenectomy for varicous veins of left leg. Episode of paraesthesia on left arm with associated transient dysartria one month ago. The CT scan of the brain failed to show brain lesions. After 2 weeks complained pain on left calf that worsened with orthostatic position and deambulation. An hyposfigmia of the left popliteal artery was present.

Recently, patient complained palpitations and chest pain that were associated with pain on the left calf at rest and also edema on physical examination.

He had normal S1 and loud pulmonary component of second heart sound, high rate (150 bpm), soft murmur on tricuspid and pulmonary focus on auscultation. Chest and lung fields were clear. Jugular veins were turgid.

Blood pressure 105/70 mmHg. Bilateral ankle edema and trophic ulcer on right medial malleolus.

**Figure 1:**
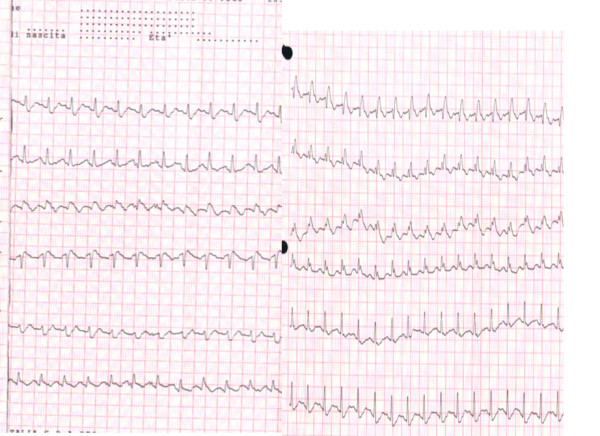
Electrocardiogram showed: supraventricular tachycardia, RBBB.

The Electrocardiogram showed: supraventricular tachycardia, RBBB (fig. 1).

**Figure 2:**
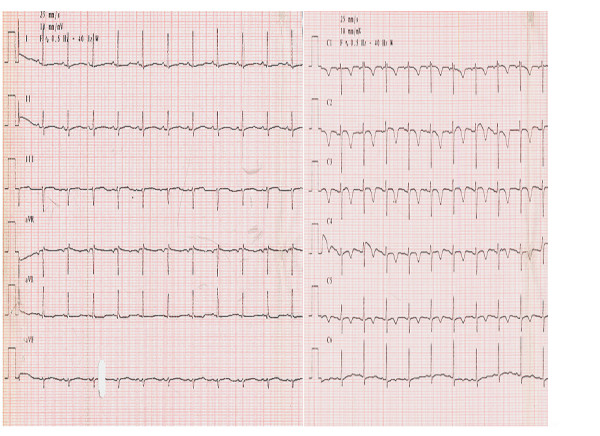
An electrocardiogram performed two hours later revealed a sinus rhythm, HR 88/min, negative T-waves on precordial leads V1, V2, V3, V4, a right ventricular strain (fig. 2).

In our department, the patient received amiodarone, unfractioned heparin infusion. An electrocardiogram performed two hours later revealed a sinus rhythm, HR 88/min, negative T-waves on precordial leads V1, V2, V3, V4, a right ventricular strain (fig. 2).

Arterial blood gas measurement showed PaO2 60 mmHg, PaCO2 36 mmHg; Dimer-Test 1650; Serum Troponin I 0.16 ng/ml; genetic screening tests for thrombophilia were negative.

Chest X-ray showed cardiomegaly, right pleural effusion. Venous duplex scanning showed no signs of either superficial or deep vein thrombosis.

**Figure 3:**
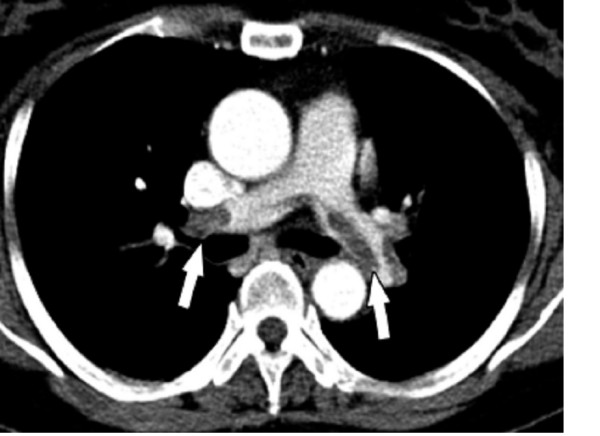
Chest Spiral TC (with and without contrast agent) showed multiples filling defects of principal branches, due to acute and chronic pulmonary embolism.

In order to avoid the presence of a pulmonary embolism, the following exams were performed: Chest Spiral TC (with and without contrast agent) showed multiples filling defects of principal branches and superior and inferior segmentary branches of pulmonary artery, due to acute and chronic pulmonary embolism. Basal parenchymal thickening was noticed (fig. 3).

Phlebography of inferior limbs and cavography pointed out no signs of deep vein thrombosis, normal deep venous circle, normal inferior cava vein, varices of left ankle communicating veins.

A transthoracic echocardiogram (TTE 4-chamber. subcostal view) was performed to evaluate the right ventricular function and the right pulmonary pressure.

Left ventricle was'nt dilated and global systolic function was normal.

**Figure 4:**
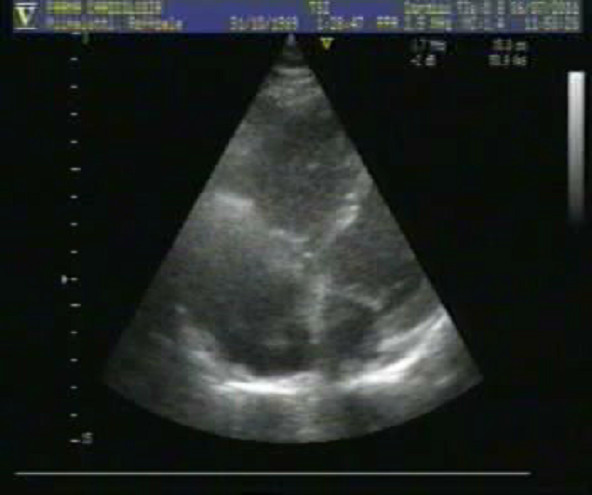
**Transthoracic echocardiogram (TTE 4-chamber)**. Right sections were dilated with a hypokinetic right ventricle. An image referring to a thrombosis was detected in the rigtht atrium.

Interventricular septum movement was diskinetic. Right sections were dilated with a hypokinetic right ventricle. Systolic pulmonary artery pressure (PAPs) was estimated 55 mmHg. An image referring to a thrombosis was detected in the rigtht atrium. (Fig 4 – additional file 1).

**Figure 5:**
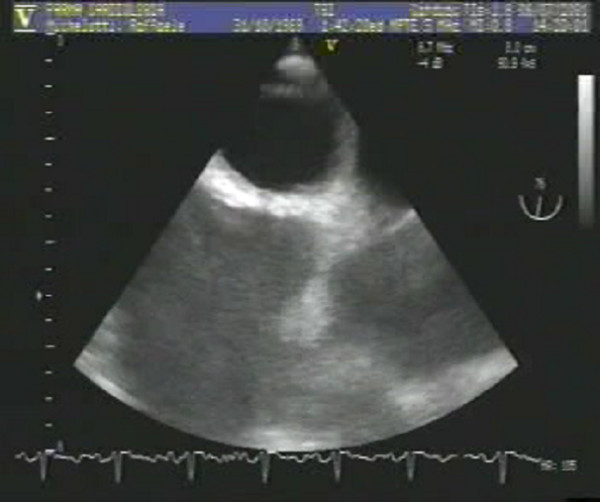
**Transesophageal echocardiogram (TEE transverse view 70°)**. Bulky and serpiginous formation referring to a thrombus that is laid through the foramen ovale and which protrudes in both atria, in burden cycles overcoming the right atrium-ventricular plane.

To evaluate the presence of a thrombus in right atrium a transesophageal echocardiogram was performed (Fig 5 – TEE transverse view 70°).

Bulky and serpiginous formation referring to a thrombus that is laid through the foramen ovale and which protrudes in both atria, in burden cycles overcoming the right atrium-ventricular plane. A patent foramen ovale was confirmed by injection of saline contrast agent. (additional file 2).

Therefore patient was referred to the surgeon and a vena cava filter was inserted.

Trough a sternal approach a right atriothomy was made and a voluminous thrombus strangled through the patent ovale foramen (PFO) was displaced after cutting the interatrial septum, which was subsequently stitched.

The intraoperative TEE excluded the presence of residual thrombus on the patent foramen ovale.

During the postoperative course the patient displaced a right ventricular failure with a low output syndrome, treated successfully with inotropic drugs.

The patient was dismissed with a long-term oral anticoagulation therapy. Moderate pulmonary hypertension was still present after 4 years (48–50 mmHg on TTE).

## Discussion

Patent foramen ovale causes a paradoxical embolism, when right pulmonary pressures are higher than normal like in the case of to relapsing pulmonary embolism or during a Valsalva. A thrombus, which has grown 'in situ'[2] or trapped through the patent foramen ovale, may be a cause of relapsing pulmonary or systemic embolism during anticoagulation therapy.

- In the present case the patient was referred to the surgery in order to avoid the risk of systemic embolism

- Current Clinical Practice Guidelines recommend the use of Chest Spiral TC for the diagnosis of pulmonary embolism and echocardiography imaging plays a role only in the unstable patients and in complex clinical cases [3,4].

- Patients with pulmonary embolism with a PFO greater than 4 mm have 10-fold risks of death and 5-fold risks of systemic embolism in regard to patients without PFO [5].

A TTE with a saline contrast agent could be used in case of pulmonary embolism and pulmonary hypertension to exclude the presence of a PFO.

- Pulmonary hypertension generally falls during the subsequent 6 weeks after a pulmonary embolism reaching a stable level successively.

- Patients with systolic pulmonary artery pressure higher than 50 mmHg at the beginning, generally develop pulmonary hypertension (HTP) [6].

- Patients with HTP and right ventricular failure have a worse prognosis at 5 years.

## Conclusion

The present case report shows a rare case of thrombus located through the PFO, in patient with pulmonary and paradoxical embolism, which takes back to the hypothesis thrombus growth.

In order to prevent recurrent paradoxical embolism, percutaneous closure of PFO is recommended, but in this case, thrombus was trapped through the PFO and the patient was referred to the surgeon.

We believe that under these circumstances the clinician should be informed of the presence of PFO in critical pulmonary embolism; his case points out the key role of TEE to face a diagnostic and therapeutic scenarios.

## Abbreviations

PFO- Patent foramen ovale.

TTE- Transthoracic echocardiography.

TEE- Transesophageal echocardiography.

HTP- Pulmonary hypertension.

## Authors' contributions

WS has performed echocardiographic examinations for this article and has prepared the manuscript. GDI has performed the literature rewiew. All the authors have approved the final review of the manuscript.

## Supplementary Material

Additional file 1 Left ventricle volume and global systolic function were normal. Interventricular septum movement was diskinetic. Right sections were dilated with a hypokinetic right ventricle. Systolic pulmonary artery pressure (PAPs) was estimated 55 mmHg. An image referring to a thrombosis was detected in the rigtht atrium. Click here for file

Additional file 2 Bulky and serpiginous formation referring to a thrombus that is laid through the foramen ovale and which protrudes in both atria, in burden cycles overcoming the right atrium-ventricular plane. A patent foramen ovale was confirmed by injection of saline contrast agent. Click here for file
